# Innovative behavior and structural empowerment among the Chinese clinical nurses: the mediating role of decent work perception

**DOI:** 10.1186/s12912-024-02554-z

**Published:** 2024-12-03

**Authors:** Zhangyi Wang, Li Yang, Yue Zhu, Xiaochun Tang, Tingrui Wang, Li Chen, Liping Li, Weimin Xie, Jiaofeng Peng, Jie Yang, Qianxiang Long, Feng Lu, Yan Wang, Huilong Shen, Jun Yin, Xiaoping Zhan, Huifang Zhou

**Affiliations:** 1 Nursing Department, Affiliated Hengyang Hospital of Hunan Normal University & Hengyang Central Hospital, No.10, Yancheng Road, Yanfeng District, Hengyang, Hunan Province 421001 China; 2 Children Respiratory, Zone 2, Chenzhou No.1 People’s Hospital, No.102 Luojiajing, Chenzhou, Hunan Province 423000 China; 3 Nursing Department, Tianjin Academy of Traditional Chinese Medicine Affiliated Hospital, Tianjin, China; 4 School of Nursing, Guizhou Medical University, Guiyang, Guizhou Province China; 5 School of Nursing, Kiang Wu Nursing College of Macao, Macao, China; 6 The Second Affiliated Hospital of University of South China, Hengyang, Hunan Province China

**Keywords:** Innovative behavior, Structural empowerment, Decent work perception, Mediating role, Clinical nurses, China

## Abstract

**Background:**

Clinical nurses play a vital role in healthcare. Their innovative behavior is crucial for improving patient care, advancing the profession, and ensuring the healthcare industry’s continued success. Many studies have highlighted the importance of nurse innovative behavior, but the link between their innovative behavior, structural empowerment, and decent work perception remains unclear.

**Objectives:**

This study aimed to investigate the relationship between innovative behavior, structural empowerment, and decent work perception among the Chinese clinical nurses and identify the mediating role of decent work perception.

**Methods:**

A cross-sectional correlational design was employed, and from July 2023 to April 2024, 1,513 clinical nurses were recruited from 8 tertiary grade-A hospitals across three cities in China. Data from the Demographic Characteristics Questionnaire, the Nurse Innovation Behavior Scale, the Conditions of Work Effectiveness Questionnaire-II, and the Decent Work Perception Scale were collected through convenience sampling and analyzed using descriptive statistics, univariate correlation, and process plug-in mediation effect analyses.

**Results:**

The total scores of innovative behavior, structural empowerment, and decent work perception were 28.36 ± 6.25, 51.15 ± 12.63, and 42.97 ± 9.25, respectively. Innovative behavior was significantly, moderately and positively correlated with structural empowerment (*r* = 0.657, *p* < 0.01) and decent work perception (*r* = 0.618, *p* < 0.01); decent work perception played a partial mediating role between innovative behavior and structural empowerment (52.5%).

**Conclusion:**

The innovative behavior, structural empowerment, and decent work perception among the Chinese clinical nurses were relatively moderate, indicating a need for improvement. Structural empowerment perception can, directly and indirectly, impact innovative behavior through decent work perception among Chinese clinical nurses. Nursing managers should promote innovative behavior of clinical nurses by raising structural empowerment and decent work perception to improve the quality of clinical nursing. Thus, it can be improved by creating a positive empowerment climate for clinical nurses and providing them with the information, resources, support, and opportunities for their jobs and improving their level of structural empowerment and decent work perception.

**Supplementary Information:**

The online version contains supplementary material available at 10.1186/s12912-024-02554-z.

## Introduction

With the emergence of high-tech smart healthcare construction, such as artificial intelligence, big data, and blockchain, nursing services also face new challenges in connotation and outreach, urgently needing innovative development. Healthcare organizations are currently experiencing a significant period of transformation driven by rapid technological advancements and shifting societal needs [1]. Clinical nurses play a crucial role in healthcare, as their innovative behavior enhances nursing quality and is vital in driving the sustainable development of the nursing discipline and healthcare industry [2]. Consequently, it is crucial for healthcare leaders, particularly nurse managers, to maintain a balance between adopting innovative approaches and enhancing existing practices to deliver high-quality care [3]. The International Council of Nursing (ICN) defines nursing innovation as systematically creating novel approaches, technologies, and work practices. This includes generating ideas and their translation into practical applications, implementation, and completion. The ICN highlights the crucial role of nursing innovation in advancing health, disease prevention, and enhancing the quality of care [4]. In addition, Bao et al.‘s [5] definition of nurses’ innovative behavior is widely accepted in China, where nurses seek and develop new methods, technologies, and working approaches to promote health, reduce diseases, and enhance the quality of patient care. Many foreign studies have proven that innovative behavior of nurse not only effectively improved the quality of nursing services and met patients’ health service needs but also played an important role in reducing the number of days patients stayed in the hospital, improving healthcare resource utilization, and controlling costs [6, 7]. In China, the National Health Planning Commission also clarified and emphasized the importance of innovation in the medical and health fields [8]. However, only a few studies were conducted on the innovative behavior of Chinese clinical nurses and its correlation with other factors. Therefore, it is essential to investigate the innovative behavior of clinical nurses and explore its relationship with other related variables.

Ample evidence indicates that many factors, including individual and environmental, influence the innovative behavior of nurses [9]. Information literacy, personal perceptions, and psychological resilience and empowerment are the individual factors that influence nurses’ innovative behavior. In addition, structural empowerment, authentic leadership, organizational support, and other external environmental factors are environmental factors that are associated with the innovative behavior of nurses [10].

### Literature review and hypotheses development

#### Structural empowerment may positively predict innovative behavior

Structural empowerment refers to the nursing managers providing a supportive and growth-oriented work and learning environment for nursing staff to access timely information, mobilize organizational resources, and receive organizational support to achieve professional goals [11]. Research shows that nurses’ perceived empowerment increases their job engagement and satisfaction and reduces burnout. Structural empowerment also provides the conditions for creating and implementing new ideas through information, resources, support, opportunities, and the informal power of interpersonal networks [12, 13]. Structural empowerment can also positively impact nurses’ professional behavior, such as innovation, leadership, professional practice behaviors, clinical decision-making skills, and inter-professional collaboration [14, 15]. Many studies in China have found that structural empowerment can directly and positively influence nurses’ individual innovative behavior and play a positive role in predicting their innovative behavior, among which information power and self-efficacy have the strongest correlation with innovative behavior [16, 17]. However, existing research suggests that clinical nurses have low levels of perceived structural empowerment [18]. Moreover, the mechanisms underlying the effect of structural empowerment on innovative behavior have not been clarified.

#### Decent work perception may positively predict innovative behavior

Decent work perception refers to an individual’s personal and inherent perception of the value and dignity of their work, which contributes to a stronger sense of organizational identity and a positive emphasis on work [19]. Securing decent work conditions for nurses is recognised as a crucial component of sustainable development within the framework of the United Nations’ 2030 Agenda (WHO 2020), underscoring the importance of it. Despite this recognition, research investigating the benefits of decent work conditions among nurses remains limited [20]. An understanding of coexistence and inclusion in both work status and interpersonal relationships fosters the fulfillment of employees’ relational needs, the recognition and respect from others in the workplace, a sense of inner satisfaction and happiness, and a sense of personal worth, which contribute to the motivation to achieve valuable work outcomes [21]. When employees’ psychological needs for autonomy and competence are met in the organization, they will be motivated to produce more innovative behavior, and a research shows decent work perception significantly impacts employees’ innovative behavior [22]. Similarly, many studies in China showed that decent work enhances physical and mental health, psychological ownership, psychological empowerment and work immersion of nurses [23, 24]. Moreover, the results of these studies on nurses in China aligns with previous nonnursing research that showed decent work increases employees’ feelings of belonging to their organization, so as to improve their innovative behavior [25]. Additionally, foreign researcher El-Gazar et al. found that decent work is a significant predictor in nurturing felt obligation and organisational identification, and securing decent work conditions, fostering felt obligation and organisational identification contribute to innovative behavior [26]. Zoromba et al. also found that there is a positive correlation between decent work conditions and the ethical ideologies of nurses, which can assist hospital administrators in fostering work conditions that promote appropriate ethical ideologies and innovation consciousness among nurses [27]. Multiple studies show that clinical nurses’ decent work perception is moderate to low and needs further improvement [24, 28, 29]. Yet, the impact of decent work perception on the innovative behavior of clinical nurses has not been clarified, and the relationship between structural empowerment, decent work perception, and innovative behavior needs to be further explored.

### Theoretical framework of this study

This study employs space theory [30, 31], which is grounded in field dynamics theory [32]. The theory states that the formula of innovative behavior is expressed by *Bi = f (Pi × Ei)*, where *Bi* represents behavior (including innovative behavior), *Pi* represents the person (encompassing internal needs, perceptions, and psychological factors), *Ei* represents the environment (including structural empowerment and external factors), and *f* is the function coefficient (a relatively fixed value). The innovation subject (*Pi*) and the innovation environment (*Ei*) are interdependent variables that collectively form the innovation space. Based on the space and field dynamics theories, nurses’ innovative behavior can be interpreted as a response to external factors disrupting their usual work routine. Nurses engage in innovative behavior to adapt to the changing environment, restore balance, and continue their nursing work.

**Fig. 1:**
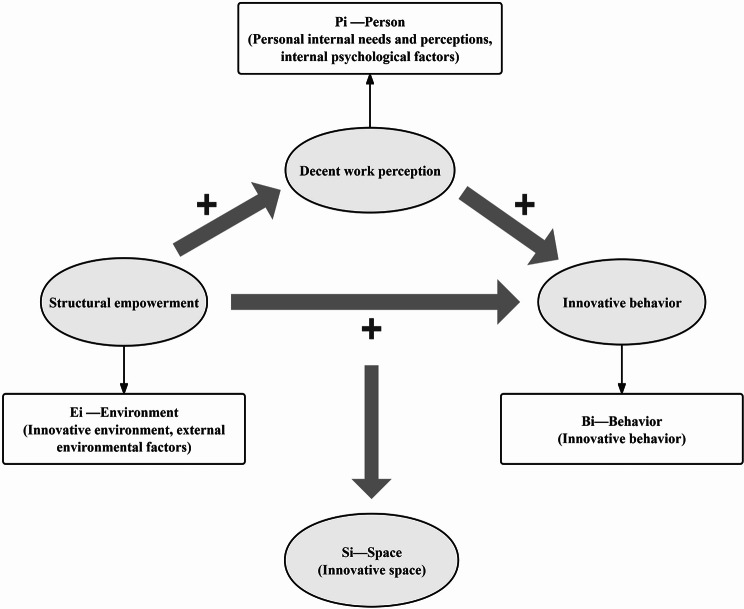
The conceptual framework among innovative behavior, structural empowerment and decent work perception of the study

In conclusion, the correlations between innovative behavior, structural empowerment, decent work perception, and their role in Chinese clinical nurses are still open for further research. According to the space theory, the internal personal factors (decent work perception in this study) and external environmental factors (structural empowerment in this study) interact with each other and ultimately contribute to the innovative behavior of clinical nurses. Thus, based on this theory and the literature review, the conceptual framework for this study was constructed, as shown in Fig. 1. Therefore, the significance of this study is to investigate the innovative behavior, structural empowerment, and decent work perception and their relationships among the Chinese clinical nurses, focusing on exploring the mediating role of decent work perception, and to provide a reference for constructing targeted intervention measures to improve structural empowerment and decent work perception, thus improving nurses’ innovative behavior and further improving nursing quality and satisfaction.

## Objectives

This study aimed to (1) investigate the innovative behavior, structural empowerment, and decent work perception among the Chinese clinical nurses; (2) examine relationships between innovative behavior, structural empowerment, and decent work perception; (3) explore the mediating role of decent work perception between innovative behavior and structural empowerment.

## Methods

### Study setting

A cross-sectional correlational design was carried out, and the study followed the Strengthening the Reporting of Observational Studies in Epidemiology Statement (STROBE).

### Participants and sample

Convenience sampling was used to recruit clinical nurses from eight tertiary grade-A hospitals (with more than 1,500 beds and 2,000 employees) across three cities in China: Tianjin and Shijiazhuang in the north, and Guiyang, Changsha, Chenzhou, and Hengyang in the south. The recruitment period was from July 2023 to April 2024, where the total population size of the study was nearly 8,500 clinical nurses. Respondents met the following criteria. Inclusion criteria: (1) an employed registered nurse; (2) working in clinical nursing for more than 3 months. Exclusion criteria: (1) intern nurses; (2) advanced training, rotation, and regular training nurses; and (3) nurses who were not on duty during the investigation period. Informed consent was taken from each nurse who volunteered to participate in the study.

To determine the minimum sample size, we employed the G*Power 3.1.9.7 software [33]. The calculation parameters for F-tests were set as follows: α error probability: 0.05; Power (1 - β error probability): 95%; Statistical test: “Linear multiple regression: Fixed model, R² deviation from zero;” Type of power analysis: “A priori: Compute required sample size - given α, power, and effect size.” The resulting minimum sample size was calculated to be 107 participants.

To enhance the generalization and external validity of the findings, we distributed a larger number of questionnaires. This approach helps minimize potential biases stemming from geographical variations and differences in hospital characteristics. These questionnaires are crucial for enhancing the transparency and understanding of the study design, particularly concerning the mediation analysis. Thus, based on the design of cross-sectional study and the requirement of large sample size, convenient sampling was used in this study. Convenient sampling is suitable for the exploratory, descriptive, and observational study and the study of obtaining a large amount of preliminary information quickly, and the principle of convenient sampling is researchers determines the unit to be included in the sample by themself and choose samples at a specific time and place according to the principle of convenience. A total of 1,535 questionnaires were distributed. After eliminating 22 questionnaires with common or obviously contradictory responses, we obtained 1,513 valid questionnaires. This resulted in an effective recovery rate of 98.6%.

### Measurements

#### The demographic characteristics questionnaire

The Demographic Characteristics Questionnaire was developed through a literature review containing 15 variables, such as gender, age, educational background, technical title, nurse experience, and so on, as shown in Table 1. The Cronbach’s α of the scale calculated in this study was 0.911.

**Table 1:** Demographic characteristics and univariate analysis of NIBS among the Chinese clinical nurses [ *n* = 1,513, M (SD) ]

Characteristics	*n*	%	Total score of NIBS		*t / F*-value	*p*-value
			M	SD		
**Gender**					*t****=*** -0.783	0.336
Male	135	8.9	27.12	8.37		
Female	1, 378	91.1	28.51	9.11		
**Age (years)**					*F* = 0.832	0.755
≤ 25	486	32.1	28.02	7.63		
26–35	693	45.8	28.33	8.15		
36–45	250	16.5	29.42	8.62		
> 45	84	5.6	28.25	9.26		
**Educational background**					*F* = 23.256	< 0.001**
Junior college degree	141	9.3	20.30	7.29		
Bachelor degree	1307	86.4	29.83	7.68		
Master degree or above	65	4.3	34.25	8.21		
**Technical title**					*F* = 19.228	< 0.001**
Nurse	269	17.8	24.25	6.85		
Nurse Practitioner	812	53.7	27.13	8.71		
Nurse-in-Charge	327	21.6	30.65	7.46		
Associate Nurse Practitioner and above	105	6.9	33.06	9.12		
**Nurse experience (years)**					*F* = 1.225	0.427
≤ 5	658	43.5	28.13	8.36		
6–10	455	30.1	28.25	8.25		
11–15	208	13.8	29.06	8.51		
16–20	125	8.2	28.40	7.98		
> 20	67	4.4	28.93	9.01		
**Per capita monthly income (RMB)**					*F* = 0.931	0.585
≤ 3000	73	4.8	27.68	7.38		
3001–5000	281	18.6	29.22	9.02		
5001–7000	443	29.3	28.17	8.16		
7001–9000	385	25.4	28.58	8.36		
> 9000	331	21.9	29.15	8.26		
**Number of night shifts per month (times)**					*F* = 3.208	0.032*
≤ 5	623	41.2	30.67	8.21		
6–10	828	54.7	27.88	9.22		
> 10	62	4.1	24.26	8.65		
**Employment modality**					*F* = 1.273	0.371
Enterprise system	478	31.6	27.58	7.72		
Contractual system	794	52.5	28.32	7.46		
Labour dispatch system	241	15.9	29.01	8.83		
**Administrative position**					*F* = 17.263	< 0.001**
None	1384	91.5	26.37	7.31		
Head nurse	79	5.2	31.47	8.25		
Head nurse of ward	38	2.5	33.59	7.88		
Director of nursing department	12	0.8	36.26	8.92		
**Hospital nature**					*t* ***=*** 3.168	0.002**
Specialized hospital	236	15.6	29.52	8.86		
General hospital	1277	84.4	26.93	9.12		
**Whether is a specialist nurses**					*t* ***=*** 12.108	< 0.001**
Yes	186	12.3	34.12	8.28		
No	1327	87.7	25.73	7.83		
**Whether is a clinical instructor**					*t* ***=*** 5.526	< 0.001**
Yes	401	26.5	30.43	8.13		
No	1112	73.5	27.06	8.32		
**Whether have ever applied for a nursing research project**					*t* ***=*** 8.528	< 0.001**
Yes	185	12.2	32.28	7.66		
No	1328	87.8	27.38	8.25		
**Whether have ever published a paper**					*t* ***=*** 9.883	< 0.001**
Yes	262	17.3	33.12	7.89		
No	1251	82.7	27.06	7.61		
**Whether have attended a nursing research programme**					*t* ***=*** 1.076	0.243
Yes	156	10.3	29.08	8.92		
No	1357	89.7	28.13	8.57		

Note M = mean, SD = standard deviation, “*t*”: independent *t*-test, “*F*”: one-way ANOVA test, *: *p* < 0.05, **: *p* < 0.01

#### The nurse innovation behavior scale (NIBS)

The NIBS was used to investigate the innovative behavior of nurses. The scale was compiled by Bao et al. [5] according to the characteristics of Chinese nurses. It includes three dimensions and 10 items of “Generating ideas” (3 items), “Obtaining support” (4 items), and “Realizing ideas” (3 items). The Cronbach’s α of the original scale is 0.879 in Bao et al.‘s study of 825 Chinese nurses, and the reliability coefficients of “Generating ideas,” “Obtaining support,” and “Realizing ideas” are 0.746, 0.834, and 0.870, respectively. The content validity index (CVI) of the total scale is 0.910, and the CVI of each dimension is 0.880–0.950, which shows that the scale has good reliability and validity. In this study, the combined Cronbach’s α of all 1,513 valid questionnaires was 0.926. Likert-5 scoring method was used, with the 1–5 scores indicating “never,” “less,” “sometimes,” “often,” and “frequently,” respectively. The total score is between 10 and 50 points, a higher score reflects higher innovative behavior.

#### The conditions of work effectiveness questionnaire-II (CWEQ-II)

The CWEQ-II was used to investigate nurses’ perceived level of structural empowerment. The scale was created by Huang et al. [34] according to the characteristics of Chinese nurses. It consists of 19 items in six dimensions: “Opportunity empowerment,” “Information empowerment,” “Resource empowerment,” “Supportive empowerment,” “Formal empowerment,” and “Informal empowerment.” The scale’s reliability and validity in metrology are demonstrated by its excellent accuracy, stability, and consistency, as evidenced by the Cronbach’s α values of 0.757–0.910 for the six dimensions included in the original scale, 0.936 for the entire scale, 0.878 for folding reliability, 0.912 for test-retest reliability, and 0.920 for CVI of 398 Chinese nurses in Huang et al.‘s study. The combined Cronbach’s α of 1,513 valid questionnaires in this study was 0.929. A 5-point Likert scale was used, with scores ranging from 19 to 95 on a scale of “1” to “5,” from “none” to “many,” higher scores indicated higher structural empowerment.

#### The decent work perception scale (DWPS)

The DWPS, developed by Mao et al. [35], was used, comprising 16 items in five dimensions: “Working rewards,” “Working position,” “Career development,” “Career recognition,” and “Working atmosphere.” The original scale has a Cronbach’s α of 0.745, and the Cronbach’s α of the five dimensions ranges from 0.720 to 0.751 in Mao et al.‘s study of 625 Chinese medical staffs. The total scale has a CVI of 0.880, indicating that the scale has good reliability and validity. This study’s total Cronbach’s α of 1,513 valid questionnaires was 0.940. The scale is based on a 5-point Likert scale, with scores from 16 to 80 on a scale of “1” to “5,” from “completely disagree” to “completely agree,” the higher the score, the higher decent work perception.

### Ethics considerations and data collection

This study has been approved by the Medical Ethics Committee of Hengyang Central Hospital (2023-031-18). First, after obtaining permission from hospital administrators, the researchers approached the participants with the help of the head nurses. Second, the researchers provided information to participants before the investigation regarding the purpose, confidentiality, consent, and questionnaire items. The questionnaires were designed to maintain anonymity and confidentiality. This was achieved by ensuring no identifying marks, names, or numbers were associated with the participants. The study participants were free to decline or withdraw at any time, and it was explained that the information collected would be used exclusively for academic research and not for profit. Once participants consented to participate, they were provided with written study information and an informed consent form to sign. The researchers assisted the participants in completing the written information, the informed consent form, and the questionnaire. During face-to-face interactions, the surveys were efficiently completed within 15–20 min. Nurses were instructed to fill out the questionnaires in person, and upon completion, the questionnaires were promptly collected and verified. In this study, 1,535 questionnaires were delivered; however, 22 questionnaires with regular or obviously contradictory answers were eliminated. Finally, 1,513 valid questionnaires with an effective recovery rate of 98.6% were obtained.

### Statistical analysis

IBM SPSS version 21 (IBM Corp., Armonk, NY, USA) was used for statistical analyses. Descriptive statistics were used to investigate the demographic characteristics of the participants (gender, age, education background, technical title, nurse experience, as such) and main study variables (innovative behavior, structural empowerment, and decent work perception). Additionally, one-way analysis of variance (ANOVA), independent *t*-tests, and Pearson correlations were used to evaluate the associations between variables. If there was any difference between a demographic characteristic and the dependent variable, the characteristic was used as a covariate in the analysis.

The PROCESS v4.1 macro and bootstrap method in SPSS were used to perform the mediation tests [36]. PROCESS v4.1 has unique advantages in mediating and regulatory effects analysis. It uses Bootstrap, a non-parametric resampling technique, to evaluate the confidence interval (CI) of indirect effects. The convenience sampling method does not require strict assumptions about data distribution, making it more robust than traditional methods. In addition, The operation of PROCESS v4.1 is relatively simple. The PROCESS v4.1 macro and bootstrap methods were used instead of Baron and Kenny’s three regression models [37] or the Sobel test [38] to evaluate the significance of the indirect effect. This is because they do not require an assumption of normal distribution.

Model 4 of the PROCESS v4.1 macro was employed to investigate the mediating role of decent work perception in the relationship between innovative behavior and structural empowerment in Chinese clinical nurses. The PROCESS v4.1 macro implements bias-corrected bootstrap confidence intervals (CIs) for the indirect effect. If bias-corrected bootstrap CIs for the indirect effect do not contain 0, an indirect effect is supported. The statistical significance level was set at *p* = 0.05.

## Results

### Demographic characteristics of participants and univariate analysis of innovative behavior

A total of 1,513 clinical nurses were included in this study: 135 (8.9%) were male, 1,378 (91.1%) were female, 486 (32.1%) aged ≤ 25, 693 (45.8%) aged 26–35, 250 (16.5%) aged 36–45, and 84 (5.6%) aged > 45. Other demographic characteristics are shown in Table 1, which also indicates the total score of innovative behavior of the clinical nurses with different educational backgrounds, technical titles, number of night shifts per month, administrative positions, hospital nature, whether is a specialist nurse, a clinical instructor, have ever applied for a nursing research project, or published a paper, had statistically significant differences (all *p* < 0.05).

### Scores of innovative behavior, structural empowerment, and decent work perception

The total score of innovation behavior was 28.36 ± 6.25, and the average NIBS score was 2.58 ± 0.92. The highest and lowest mean entry scores in the three dimensions were “Generating ideas” (3.07 ± 0.71) and “Realizing ideas” (2.61 ± 0.65), respectively.

The total score of structural empowerment was 51.15 ± 12.63, and the average CWEQ-II score was 2.58 ± 0.92. The highest mean score for entries in the six dimensions was “Opportunity empowerment” (3.08 ± 0.82), and the lowest was “Information empowerment” (2.62 ± 0.71).

The total score of decent work perception was 42.97 ± 9.25, and the average DWPS score was 2.58 ± 0.92. The highest mean score for entries across the five dimensions was “Working atmosphere” (2.85 ± 0.86), and the lowest was “Working position” (2.33 ± 0.69). The NIBS, CWEQ-II, and DWPS scores are shown in Table 2.

**Table 2:** The scores of NIBS, CWEQ-II and DWPS among the Chinese clinical nurses [ *n* = 1,513, M (SD) ]

Dimensions	Number of items	Dimensional score		Average score of items		Ranking
		M	SD	M	SD	
**NIBS total score**	10	28.36	6.25	2.84	0.66	—
Generating ideas	3	9.21	2.61	3.07	0.71	1
Obtaining support	4	11.32	3.12	2.83	0.68	2
Realizing ideas	3	7.83	2.68	2.61	0.65	3
**CWEQ-II total score**	19	51.15	12.63	2.69	0.75	—
Opportunity empowerment	3	9.24	2.82	3.08	0.82	1
Information empowerment	3	7.86	2.59	2.62	0.71	6
Supportive empowerment	3	8.58	2.68	2.86	0.79	3
Resource empowerment	3	8.19	2.61	2.73	0.73	5
Formal empowerment	3	8.43	2.73	2.81	0.76	4
Informal empowerment	4	8.85	3.58	2.95	0.78	2
**DWPS total score**	16	42.97	9.25	2.69	0.68	—
Working rewards	4	10.72	3.21	2.68	0.75	4
Working position	3	6.99	2.58	2.33	0.69	5
Career development	3	8.28	2.73	2.76	0.72	3
Career recognition	3	8.43	2.62	2.81	0.83	2
Working atmosphere	3	8.55	2.67	2.85	0.86	1

Note M = mean, SD = standard deviation

### Relationships between innovative behavior, structural empowerment, and decent work perception

The results of Pearson correlation analysis showed that innovative behavior was moderately and positively correlated with structural empowerment (*r* = 0.657, *p* < 0.01), and the scores between all the dimensions were positively correlated (*r* = 0.558–0.641, all *p* < 0.01). There was also a moderate and positive correlation between innovative behavior and decent work perception (*r* = 0.618, *p* < 0.01), and the scores between all the dimensions were positively correlated (*r* = 0.516–0.592, all *p* < 0.01; Table 3).

**Table 3:** The relationships among innovative behavior, structural empowerment, and decent work perception of the Chinese clinical nurses (*n* = 1,513, *r*)

Item	1	1.1	1.2	1.3	2	2.1	2.2	2.3	2.4	2.5	2.6	3	3.1	3.2	3.3	3.4	3.5
**1 NIBS total score**	—																
1.1 Generating ideas	0.913**	—															
1.2 Obtaining support	0.908**	0.902**	—														
1.3 Realizing ideas	0.896**	0.885**	0.879**	—													
**2 CWEQ-II total score**	0.657**	0.641**	0.652**	0.633**	—												
2.1 Opportunity empowerment	0.613**	0.603**	0.605**	0.611**	0.883**	—											
2.2 Information empowerment	0.661**	0.620**	0.637**	0.615**	0.876**	0.865**	—										
2.3 Supportive empowerment	0.634**	0.632**	0.641**	0.623**	0.854**	0.847**	0.838**	—									
2.4 Resource empowerment	0.628**	0.613**	0.615**	0.627**	0.844**	0.802**	0.888**	0.848**	—								
2.5 Formal empowerment	0.625**	0.639**	0.636**	0.643**	0.812**	0.807**	0.825**	0.867**		—							
2.6 Informal empowerment	0.594**	0.558**	0.589**	0.576**	0.825**	0.898**	0.893**	0.858**			—						
**3 DWPS total score**	0.618**	0.613**	0.601**	0.596**	0.603**	0.599**	0.603**	0.595**	0.607**	0.596**	0.583**	—					
3.1 Working rewards	0.587**	0.565**	0.541**	0.571**	0.543**	0.568**	0.555**	0.607**	0.587**	0.565**	0.524**	0.827**	—				
3.2 Working position	0.592**	0.583**	0.536**	0.549**	0.563**	0.537**	0.521**	0.585**	0.551**	0.523**	0.538**	0.838**	0.822**	—			
3.3 Career Development	0.568**	0.541**	0.518**	0.535**	0.508**	0.522**	0.511**	0.531**	0.538**	0.542**	0.536**	0.869**	0.817**	0.825**	—		
3.4 Career recognition	0.573**	0.534**	0.517**	0.553**	0.487**	0.549**	0.517**	0.568*	0.562**	0.531**	0.533**	0.871**	0.863**	0.848**	0.851**	—	
3.5 Working atmosphere	0.578**	0.554**	0.583**	0.516**	0.521**	0.576**	0.542**	0.557**	0.578**	0.552**	0.557**	0.825**	0.855**	0.861**	0.837**	0.829**	—

Note **: *p* < 0.01, —: *r* = 1

### Mediating effect of decent work perception between innovative behavior and structural empowerment

**Fig. 2:**
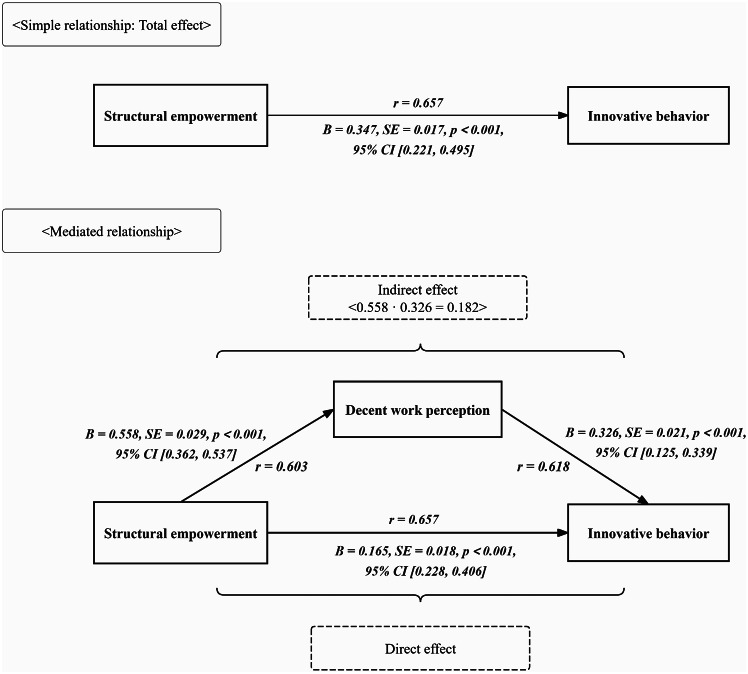
The direct effect, indirect effect and total effect among innovative behavior, structural empowerment and decent work perception of the study

The total impact of structural empowerment on innovative behavior was 0.347 (*p* < 0.01, 95% CI: 0.221–0.495). The direct effect of structural empowerment on innovative behavior was 0.165 (*p* < 0.01, 95% CI: 0.228–0.406). The indirect effect of decent work perception on innovative behavior was calculated as 0.558 × 0.326 = 0.182, accounting for 52.5% of the total effect value of 0.347 (*p* < 0.01). The bootstrapped CI ranged between 0.125 and 0.339, excluding 0, indicating that the difference was statistically significant (*p* < 0.05, as shown in Table 4; Fig. 2).

**Table 4:** The mediating effect of decent work perception between innovative behavior and structural empowerment among the Chinese clinical nurses (*n* = 1,513)

Model pathways	Standardized effect (B)	*SE*	*t*-value	*p*-value	*F*	*R*	*R* ^2^	95% *Cl*
**Total effect**					328.125	0.558	0.311	
Structural empowerment → Innovative behavior	0.347	0.017	20.412	< 0.001**				[0.221, 0.495]
**Direct effect**					263.418	0.492	0.242	
Structural empowerment → Decent work perception	0.558	0.029	19.241	< 0.001**				[0.362, 0.537]
Structural empowerment → Innovative behavior	0.165	0.018	9.167	< 0.001**				[0.228, 0.406]
Decent work perception → Innovative behavior	0.326	0.021	15.524	< 0.001**				[0.125, 0.339]
**Indirect effect**					—	—	—	
Structural empowerment → Decent work perception → Innovative behavior	0.182	0.012	—	—				[0.153, 0.356]

Note **: *p* < 0.01

## Discussion

### Status quo of the innovative behavior, structural empowerment, and future work self

The results of this study showed that the total score of innovative behavior was 28.36 ± 6.25 and the average score was 2.84 ± 0.66, which was moderate and similar to the results of Wang et al. [8] for the clinical first-line nurses during the Omicron pandemic. This is because the nurses in this study were mostly from tertiary grade-A hospitals, where the demanding clinical nursing duties and the shortage of nursing staff resulted in a lack of motivation for nurses to adhere to traditional rules and procedures, thereby restricting their innovative behavior. However, compared to ordinary nurses, clinical front-line nurses experience a greater psychological burden during the epidemic, and the traditional nursing work mode and methods cannot meet the needs of the epidemic situation. Therefore, clinical frontline nurses will actively explore innovative ideas, improve their work mode, update new technologies and methods, improve work efficiency and nursing quality to meet the requirements of epidemic prevention and control, better serve patients, and lead to better innovative behavior. However, the study of El-Gazar et al. [39] found that indicate that nurses’ behavioral intention to accept service robots in healthcare settings is moderate, which need to be further improved, and their acceptance is influenced by various factors related to their concerns about robots, roles and competencies, and potential benefits they could gain. This also reflects that nurses’ willingness to accept new technology is not high at present, and their sense of innovation needs to be strengthened, which also reflects the results of this study.

In addition, the dimension with the highest score was “Generating ideas,” which was consistent with the results of Qiao et al. [40], indicating that clinical nurses have a certain innovative sense and can generate innovative ideas in their work. One possible reason is that universities in China are increasingly prioritizing the scientific research and innovation capabilities of nursing students due to the development of the nursing discipline and the evolving concept of nursing education. Moreover, the academic level of nurses recruited in recent years has steadily increased, and hospitals and nursing managers are increasingly emphasizing the innovative capabilities of nurses; consequently, nurses in tertiary hospitals can identify issues in their work and devise solutions [41]. The dimension that received the lowest score was “Realizing ideas,” which aligns with the findings of Zhu et al. [42]. This suggests that clinical nurses primarily generate ideas for innovation but face challenges in accessing necessary information and obtaining support, hindering the actualization of their innovative ideas. This may be because most clinical nurses in this study have a bachelor’s degree. While they possess a basic understanding of nursing research, they have not conducted a systematic analysis and possess a limited capacity to analyze the feasibility of methods and retrieve literature. This impedes their innovative behavior with a heavy workload, insufficient funding for nursing research in hospitals, and a lack of time and energy [43].

The total score of structural empowerment was 51.15 ± 12.63, and the average score was 2.69 ± 0.75, which was similar to the result of Kang et al. [44]. The dimension with the highest score was “Opportunity empowerment,” and the lowest score was for “Information empowerment.” The above results indicate that hospitals can provide working conditions that facilitate learning and improvement [14]. However, clinical nurses do not have enough knowledge and information to do their jobs. They have a heavy workload, are tired of completing their daily work within a limited time frame, and cannot pay enough attention to relevant information about the hospital’s development [12]. Thus, it is recommended that nursing managers actively facilitate open lines of authority for nurses to access various resources, improve communication of hospital meeting content, and utilize the Internet to disseminate hospital-related information to clinical nurses. This will ensure that nurses have the necessary access to information, support, resources, and development opportunities to effectively carry out their work and enhance their level of structural empowerment.

The total score of decent work perception was 42.97 ± 9.25, and the average score was 2.69 ± 0.68, which was similar to the result of Jiang et al. [29] on Chinese pediatric nurses. The dimension that received the highest score was “Working atmosphere,” suggesting that clinical nurses are more content with the working environment and perceive greater respect, importance, humanistic care, and overall positivity within the organization. The lowest score was for “Working position,” indicating that the working conditions of clinical nurses need to be improved as the workload is high. The increasing incidence of various diseases, rising number of hospital admissions, and growing demand for medical resources due to China’s aging population and environmental changes have led to a higher burden of medical care; consequently, hospitals have become crowded and noisy, and nurses are facing an increasingly heavy workload [22, 45]. Therefore, it is suggested that hospital managers be prompted to strengthen the promotion of mobile healthcare and transform in-hospital treatment into patient-centered home healthcare to reduce the work pressure on healthcare workers. Simultaneously, they should embrace a magnetic hospital management model, enhance the nursing work environment, minimize the nurses’ workload, and effectively motivate them to elevate their decent work perception.

### Positive relationships between innovative behavior, structural empowerment, and decent work perception

A moderately positive correlation was observed between innovative behavior and structural empowerment (*r* = 0.657, *p* < 0.01); the higher the structural empowerment, the higher the innovative behavior of clinical nurses, which was similar to the result of El-Gazar et al. [46] on nurses working at three hospitals in Port Said. The study suggests that enhancing the ambidexterity of nurse leaders can foster a sense of psychological safety, improving their level of structural empowerment, which, in effect, contributes to increased creativity among nurses. Previous studies have shown that a supportive environment can lead clinical nurses to perceive the value and meaning of their work and increase their willingness to tackle challenging and creative tasks [47]. Nurses with higher structural empowerment have access to more information, resources, and opportunities for growth and advancement to actively implement innovative practices, thus promoting the output of innovative behavior [13]. Besides, in an environment with higher structural empowerment, nurses are more inclined to actively participate in team innovation activities and help other colleagues, and colleagues who receive help will be grateful for positive feedback [15]. Therefore, nursing managers should create a positive empowerment climate for clinical nurses by providing them with the information, resources, support, and opportunities for their jobs and improving their level of structural empowerment, which in turn promotes the output of innovative behavior.

Additionally, the study found that innovative behavior was moderately and positively correlated with decent work perception (*r* = 0.618, *p* < 0.01); the higher the work perception, the higher the innovative behavior of clinical nurses, which was similar to the result of El-Gazar et al. on 289 nurses. According to resource conservation theory, individuals with sufficient resources will try to cultivate a value-added resource spiral to increase the stock of resources [48]. Nurses with a high level of decent work perception tend to have access to more labor resources and better conditions [29]. To keep their resources and increase their stock of resources, nurses are more willing to invest their existing resources in activities that can bring a return on resources. Nurses who participate in innovative activities receive recognition and appreciation from their superiors or the organization and thus receive more resources [30]. Therefore, it is suggested that nursing administration pay attention to the importance and improvement of decent work perception, create a safe and harmonious departmental atmosphere, explore ways to enhance nurses’ value, actively guide nurses in career planning, create more opportunities for career advancement and development for nurses, and improve nurses’ sense of professional identity. Besides, nursing administration should also value decent work practices, which could enhance psychological ownership, resulting in a potential improvement in nurses’ vigor at work. This will, in turn, promote the output of innovative behavior.

### Mediating role of decent work perception between innovative behavior and structural empowerment

The results demonstrated that decent work perception partially mediates between innovative behavior and structural empowerment, with an effect of 52.5% (*p* < 0.01), which was similar to the result of El-Gazar et al. [49]. This suggests that structural empowerment promotes innovative behavior and increases decent work perception. El-Gazar et al. [49] found that decent work was significantly associated with psychological ownership and vigor at work. Psychological ownership partially mediated the relationship between decent work and nurses’ vigor at work. This also verifies the results of this study. Another study shows that decent work for individuals depends largely on the organization’s ability to provide sufficient resources to ensure that their needs are highly satisfied in all areas [21]. When nurses perceive that the organization empowers them, they have greater control over their work; their core competencies improve, their self-confidence increases, and their decent work perception is enhanced. According to the triadic interaction model of social cognitive theory, behavior, cognition, and environment interact, with the individual’s cognition and psychology mediating between the environment and the individual’s behavior [50]. Therefore, when clinical nurses have high structural empowerment, their decent work perception is also improved, promoting their innovative behavior [51]. Hence, it is recommended that nursing administration should pay attention to the influence of nurses’ structural empowerment and decent work perception on innovative behavior, actively take targeted measures to improve innovation awareness and cognition, resulting in a potential improvement in nurses’ vigor innovative behavior and at work.

### Strengths and limitations

This study is innovative because the relationships between innovative behavior, structural empowerment, decent work perception, and their mediating roles in Chinese clinical nurses have not been thoroughly investigated. This study explores the impact of structural empowerment and decent work perception on innovative behavior based on the space theory. Furthermore, it offers a theoretical foundation for developing intervention strategies to enhance the innovative behavior and capability of Chinese clinical nurses, thereby enhancing nursing quality and satisfaction.

There were some limitations in this study. (1) The study used convenience sampling, enrolling only 1,513 Chinese clinical nurses from eight hospitals. This may result in unrepresentative samples and limited, one-sided findings that cannot be generalized. Therefore, random sampling should be used, and a multi-center, large-sample research also should be carried out in the future to enhance the generalizability of findings. (2) The study’s reliance on face-to-face interviews may have introduced social desirability bias or other factors that could affect the validity of the data. Therefore, in the process of data collection, we should strictly follow the requirements to ensure the accuracy and effectiveness of quality control. (3) Multiple linear regression analysis was not used to determine the factors influencing nurses’ innovative behavior in the study. In future studies, additional research should be conducted to develop a more rigorous design involving a larger sample of clinical first-line nurses from various regions. Furthermore, future research should investigate the factors influencing innovation behavior among clinical nurses. (4) The research method is limited to quantitative research and cross-sectional study. Investigators should conduct qualitative and longitudinal research to examine how structural empowerment and decent work perception influence innovation over time would be beneficial. And to improve the accuracy and comprehensive references for constructing intervention programs that promote innovative behavior in clinical nurses. (5) Given the increasing use of technology in healthcare, future research could explore how the integration of service robots or digital tools influences empowerment and innovation.

## Conclusion

The results of this study demonstrate that innovative behavior, structural empowerment, and decent work perception among Chinese clinical nurses needed improvement. Innovative behavior was positively correlated with structural empowerment and decent work perception, and decent work perception partially mediated the change in innovative behavior and structural empowerment. Nursing supervisors are advised to improve clinical nurses’ decent work perception and innovative behavior by increasing their structural empowerment, enhancing the quality of care, and promoting the development of nursing discipline.

## Electronic supplementary material

Below is the link to the electronic supplementary material.

Supplementary Material 1

Supplementary Material 2

Supplementary Material 3

Supplementary Material 4

## Data Availability

The datasets generated and/or analyzed during the this study are not publicly available due to the privacy of the participants, but the relevant data of this study can be obtained from the first author or corresponding author on reasonable request.
