# Supplementary material for: Innovative behavior and structural empowerment among the Chinese clinical nurses: the mediating role of decent work perception
Source: BMC Nurs. 2024 Dec 3;23:881. doi: 10.1186/s12912-024-02554-z (PMC11613594; doi:10.1186/s12912-024-02554-z)
Supplement: Supplementary file 2 — Supplementary Material 2 [file 12912_2024_2554_MOESM2_ESM.docx]

**Conflict of Interest Statement**

The all authors declare that there are no conflicts of interest regarding the publication of this article.

To be signed by all authors.

For all manuscripts, please answer the following questions for each author.

1. Are the author’s university, institutional and corporate affiliations included in the title page? Yes √ No____

2. Are all the applicable sources of funding for the project included in the title page? Yes √ No____

3. For each author, is the answer to any of the following questions “yes”?

a. Have you received a financial contribution from a company or organization that might benefit (or lose) financially from the results, conclusions or discussion presented in your paper/letter? Have you received funds for research (other than that listed in acknowledgment 2 above)? (examples below)

• royalties

• patent (or patent pending)

• a fee for consulting

• a fee for speaking

• funds for a member of your staff

- a fee for recruitment of research subjects or participants
- funding for travel

Yes* No__√ _

b. Do you own stocks, shares or have options in a company or organization that might benefit (or lose) financially from the results, conclusions, or discussion presented in your paper/letter?

Yes* _____ No __√ _

c. Do you have any other competing financial interests to disclose?

Yes* ____ No__√ _

*If yes, please draft and attach a conflict of interest statement that might be published as a footnote with the article. For example, “KR has been paid a consulting fee by XYZ Company.”

Although we are emphasizing financial disclosure, each author may choose to disclose other potential conflicts, which could include an academic association or antagonism with someone whose interest might be affected by your publication, membership in a special interest group whose interests might be affected by your paper, or other strong convictions that might have affected what you wrote.

Declaration of other potential conflicts: The all authors declare that there is no conflict of interest.

Name (print) Zhangyi Wang, Li Yang, Yue Zhu, Xiaochun Tang, Tingrui Wang, Li Chen, Liping Li, Weimin Xie, Jiaofeng Peng, Jie Yang, Qianxiang Long, Feng Lu, Yan Wang, Huilong Shen, Jun Yin, Xiaoping Zhan*, Huifang Zhou*

Date November 21^th^, 2024 MS#_________________

Article Title Innovative behavior and structural empowerment among the Chinese clinical nurses: The mediating role of decent work perception

(Adapted from Johnston, K. W., & Rutherford, R. B. (1999). Disclosure of competition of interest. *Journal of Vascular Surgery, 30,* 200-202)
