# Supplementary material for: Innovative behavior and structural empowerment among the Chinese clinical nurses: the mediating role of decent work perception
Source: BMC Nurs. 2024 Dec 3;23:881. doi: 10.1186/s12912-024-02554-z (PMC11613594; doi:10.1186/s12912-024-02554-z)
Supplement: Supplementary file 3 — Supplementary Material 3 [file 12912_2024_2554_MOESM3_ESM.pdf]

Medical Ethics Committee of Hengyang Central Hospital

Approval of Ethical Review

Ethical review batch number	2023-031-18		
Project name	Innovative behavior and structural empowerment among the Chinese clinical nurses: The mediating role of decent work perception		
Project Source (Clinical Approval)	Health Research Project of Hunan Provincial Health Commission (CN) [Grant numbers: W20243278], Hengyang Science and Technology Plan Project (CN) [Grant numbers 202222035776], etc.		
Sponsor	Affiliated Hengyang Hospital of Hunan Normal University & Hengyang Central Hospital		
Responsible institution for research	Affiliated Hengyang Hospital of Hunan Normal University & Hengyang Central Hospital		
Participating institution for research	Affiliated Hengyang Hospital of Hunan Normal University & Hengyang Central Hospital, Tianjin University of Traditional Chinese Medicine, Guizhou Medical University, The Second Affiliated Hospital of University of South China, etc.		
Principal investigator (applicant)	Zhangyi Wang		
Review category	Initial review	Review mode	Quick review
Review date:	April 18th, 2023		
Review place	Medical Ethics Committee of Hengyang Central Hospital		
Review documents:			
Study protocol version	1.0	Scheme version date	April 13th, 2023
Informed consent version number	1.0	Informed consent version data	April 13th, 2023
Others	Initial review application form<br>Study protocol (1.0/20230413)<br>Informed consent, Resumes of main researchers<br>List of participants in the project		

Review opinions:

According to the Measures for Ethical Review of Biomedical Research Involving People (2016) issued by the Ministry of Health, the Quality Management Standard for Clinical Trials of Drugs (2020) issued by the State Food and Drug Administration, the Quality Management Standard

for Clinical Trials of Medical Devices (2016), the WMA Declaration of Helsinki (2013), the CIOMS International Ethical Guide for Health-related Research Involving People (2008), the ethical principles of the Guiding Principles of Ethical Market Investigation of Drug Clinical Trials (2010), and the Management Standard for Ethical Review of Clinical Research of Traditional Chinese Medicine (2010) were reviewed by this Ethics Committee and agreed to carry out this research according to the approved clinical research plan and informed consent.			
Annual/periodic follow-up review frequency	36 months	Valid until	April 13th, 2026
Signature of chairman	Dianxiang Tan<br>April 18th, 2023	Medical Ethics Committee of Hengyang Central Hospital (Seal)	
Contacts	Dianxiang Tan	Contact information	0734-8275743
