# Supplementary material for: Innovative behavior and structural empowerment among the Chinese clinical nurses: the mediating role of decent work perception
Source: BMC Nurs. 2024 Dec 3;23:881. doi: 10.1186/s12912-024-02554-z (PMC11613594; doi:10.1186/s12912-024-02554-z)
Supplement: Supplementary file 4 — Supplementary Material 4 [file 12912_2024_2554_MOESM4_ESM.docx]

**Informed Consent Form**

Dear clinical nurses,

Hope all is well with you!

First of all, thank you very much for taking time out of your busy schedule to fill out our questionnaire. The questionnaire consists of four sections. Please read the following contents carefully. If you have any questions or questions, please ask the researcher carefully.

The aims of this study are to investigate the relationship between innovative behavior, structural empowerment, and decent work perception among the Chinese clinical nurses, focusing on identifying the mediating role of decent work perception.

This study adopts questionnaire survey method. If you agree to participate in the study, the researchers will distribute questionnaires to you during the study. Please fill in the questionnaire truthfully according to your actual situation and ask the researchers in time if you don't understand the questions.

You voluntarily decide whether to participate in this study, and whether you participate in this study or not, you will not be affected in any way. All the information you provide is only used for this study, and it is kept safely, and will not be disclosed and made public to anyone without your permission. Thank you for your cooperation! During the research, if you have any questions, please feel free to consult the relevant personnel, and we will do our best to help you.

Wish: good health and all the best!

**Signature of researcher:**

**Date:**

The researcher has explained the research related matters to me, and I have made clear the purpose, process and significance of the research. I agree to participate in this research and sign here!

**Signature of participants:**

**Date:**

**1. First Section: The Demographic Characteristics Questionnaire (Note: Tick "√" in the box that best suits your situation.)**

| **Characteristics** | **Tick "√"** | **Characteristics** | **Tick "√"** |
| --- | --- | --- | --- |
| **Gender** |  | **Administrative position** |  |
| Male | □ | None | □ |
| Female | □ | Head nurse | □ |
| **Age (years)** |  | Head nurse of ward | □ |
| ≤ 25 | □ | Director of nursing department | □ |
| 26 ~ 35 | □ | **Employment modality** |  |
| 36 ~ 45 | □ | Enterprise system | □ |
| > 45 | □ | Contractual system | □ |
| **Education background** |  | Labour dispatch system | □ |
| Junior college degree | □ | **Hospital nature** |  |
| Bachelor degree | □ | Specialized hospital | □ |
| Master degree or above | □ | General hospital | □ |
| **Technical title** |  | **Whether is a specialist nurses** |  |
| Nurse | □ | Yes | □ |
| Nurse Practitioner | □ | No | □ |
| Nurse-in-Charge | □ | **Whether is a clinical instructor** |  |
| Associate Nurse Practitioner and above | □ | Yes | □ |
| **Number of night shifts per month (times)** |  | No | □ |
| ≤ 5 | □ | **Whether have ever applied for a nursing research project** |  |
| 6 ~ 10 | □ | Yes | □ |
| > 10 | □ | No | □ |
| **Nursing age (years)** |  | **Whether have ever published a paper** |  |
| ≤ 5 | □ | Yes | □ |
| 6 ~ 10 | □ | No | □ |
| 11 ~ 15 | □ | **Whether have attended a nursing research programme** |  |
| 16 ~ 20 | □ | Yes | □ |
| > 20 | □ | No | □ |
| **Per capita monthly income (RMB)** |  |  |  |
| ≤ 3000 | □ |  |  |
| 3001 ~ 5000 | □ |  |  |
| 5001 ~ 7000 | □ |  |  |
| 7001 ~ 9000 | □ |  |  |
| > 9000 | □ |  |  |

**2. Second Section: The Nurse Innovative Behaviour Scale (NIBS) (Note: Tick "√" in the box that best suits your situation.)**

| Items | Never | Less | Sometimes | Often | Frequently |
| --- | --- | --- | --- | --- | --- |
| **Generating ideas** | | | | | |
| 1.Generate the will to solve the problem. | □ | □ | □ | □ | □ |
| 2.Method of solving problems by using resource query. | □ | □ | □ | □ | □ |
| 3.Analyze the feasibility of solving problems in practical work. | □ | □ | □ | □ | □ |
| **Obtaining support** | | | | | |
| 4.Seek the approval, support and participation of colleagues or leaders. | □ | □ | □ | □ | □ |
| 5.Investigate the new method to get more information. | □ | □ | □ | □ | □ |
| 6.Seek financial support for new methods. | □ | □ | □ | □ | □ |
| 7.Formulate specific implementation plans for the new method. | □ | □ | □ | □ | □ |
| **Realizing ideas** | | | | | |
| 8.Apply the implementation scheme to work. | □ | □ | □ | □ | □ |
| 9.Revise the implementation plan and apply it in work. | □ | □ | □ | □ | □ |
| 10.Evaluate the effectiveness of new methods regularly. | □ | □ | □ | □ | □ |

**3. Third Section: The Conditions of Work Effectiveness Questionnaire-II (CWEQ-II) (Note: Tick "√" in the box that best suits your situation.)**

| Items | None | A little | Some | More | Many |
| --- | --- | --- | --- | --- | --- |
| **Opportunity empowerment** | | | | | |
| 1.I have the opportunity to get a challenging job. | □ | □ | □ | □ | □ |
| 2.I have the opportunity to acquire new skills and knowledge at work. | □ | □ | □ | □ | □ |
| 3.I have the opportunity to apply what I have learned. | □ | □ | □ | □ | □ |
| **Information empowerment** | | | | | |
| 4.I can get information about the current situation of the hospital. | □ | □ | □ | □ | □ |
| 5.I can get information about the values of senior leaders. | □ | □ | □ | □ | □ |
| 6. I can get information about the goals of the senior leaders of the hospital. | □ | □ | □ | □ | □ |
| **Supportive empowerment** | | | | | |
| 7. I can get specific information that will help me finish my work well. | □ | □ | □ | □ | □ |
| 8. I can get specific advice on improving my work. | □ | □ | □ | □ | □ |
| 9. I can get advice to solve the problem. | □ | □ | □ | □ | □ |
| **Resource empowerment** | | | | | |
| 10. I can get enough time to do paperwork. | □ | □ | □ | □ | □ |
| 11.I can get enough time to finish the required work. | □ | □ | □ | □ | □ |
| 12.I can get temporary help when I need it. | □ | □ | □ | □ | □ |
| **Formal empowerment** | | | | | |
| 13. This hospital rewards employees for their innovation in their work. | □ | □ | □ | □ | □ |
| 14. My job is flexible in this hospital. | □ | □ | □ | □ | □ |
| 15. People know what I do at work. | □ | □ | □ | □ | □ |
| **Informal empowerment** | | | | | |
| 16. I have the opportunity to cooperate with doctors to care for patients. | □ | □ | □ | □ | □ |
| 17. My colleague asked me to help him (or her) solve work-related problems. | □ | □ | □ | □ | □ |
| 18. The leader asked my advice when solving the problem. | □ | □ | □ | □ | □ |
| 19. I have the opportunity to get advice from professionals (such as physiotherapists, occupational therapists, nutritionists) instead of just doctors. | □ | □ | □ | □ | □ |

**4. Fourth Section: The Decent Work Perception Scale (DWPS) (Note: Tick "√" in the box that best suits your situation.)**

| Items | Completely disagree | Disagree | Not sure | Agree | Completely agree |
| --- | --- | --- | --- | --- | --- |
| **Working rewards** | | | | | |
| 1.My work income is sufficient to meet the basic needs. | □ | □ | □ | □ | □ |
| 2.Compared with the local average income level, my work income is very high. | □ | □ | □ | □ | □ |
| 3.In recent years, my work income has increased as expected. | □ | □ | □ | □ | □ |
| 4.The company provided me with generous benefits (paid vacation, holiday gifts, etc.). | □ | □ | □ | □ | □ |
| **Working position** | | | | | |
| 5.My workload is so heavy that I am often in an overloaded state. | □ | □ | □ | □ | □ |
| 6.I need to work overtime frequently. | □ | □ | □ | □ | □ |
| 7.My workplace environment is crowded, noisy or dull. | □ | □ | □ | □ | □ |
| **Career development** | | | | | |
| 8.In recent years, my position or rank promotion has not met my expectations. | □ | □ | □ | □ | □ |
| 9.I feel that the promotion path in this unit is clear. | □ | □ | □ | □ | □ |
| 10.I got job-related training in my unit. | □ | □ | □ | □ | □ |
| **Career recognition** | | | | | |
| 11.My current work unit and/or occupation make me look good in front of my relatives and friends. | □ | □ | □ | □ | □ |
| 12.My current work unit and/or position make me look good in front of my peers. | □ | □ | □ | □ | □ |
| 13.My relatives, friends and colleagues are envious of my present job. | □ | □ | □ | □ | □ |
| **Working atmosphere** | | | | | |
| 14.My colleagues at work respect me very much. | □ | □ | □ | □ | □ |
| 15.I feel that my boss/supervisor cares about my personal situation | □ | □ | □ | □ | □ |
| 16.The working atmosphere of the unit is positive. | □ | □ | □ | □ | □ |
